# A Global Champion for Health—WHO’s Next?

**DOI:** 10.1371/journal.pmed.1002059

**Published:** 2016-06-28

**Authors:** The PLOS Medicine Editors

**Affiliations:** The *PLOS Medicine* Editors are Clare Garvey, Thomas McBride, Linda Nevin, Larry Peiperl, Amy Ross, Paul Simpson, and Richard Turner.

## Abstract

In this month’s editorial, the *PLOS Medicine* Editors propose ideal qualities for the World Health Organization's next Director General, for whom the selection process is now underway.

The World Health Organization (WHO) should not only be a force for good but a source of pride. Since the organization’s inception in 1948, landmark achievements in health have included the eradication of smallpox and the well-advanced, but still ongoing, project to consign poliomyelitis to history. The body’s normative functions are needed to prepare for and deploy appropriate responses to incipient health threats, notably those wrought by infectious diseases outbreaks, and underpin continuing tasks, for instance that of bringing the HIV epidemic under control. Transformational campaigns led by WHO have included the Framework Convention on Tobacco Control, enacted in 2005, a treaty that prioritized the enormous health benefits of smoking cessation for the world’s population. In drafting this Editorial, we sought opinions from members of *PLOS Medicine*’s editorial board and others, and Margaret E. Kruk, of the Harvard T.H. Chan School of Public Health, noted that “WHO is…seen by most as embodying our shared global health aspirations. Because of this its pronouncements carry great weight.”

Given WHO’s global footprint and responsibilities in addressing the many serious threats to health in diverse settings, the organization does not lack critics. This sprawling empire of more than 7,000 employees, headquartered in Geneva, Switzerland, has a presence in 150 of the organization’s 194 constituent countries, along with six regional offices, and has a bureaucracy and running costs to match. As global health has grown in the public consciousness in recent decades, WHO has come to work alongside, and invite comparisons with, some tightly focused and handsomely funded newer organizations that lack the explicit public accountability of a United Nations agency. Often, communicators about health are prolific and strident in their criticism of existing structures and organizations, especially WHO. Owing to its complex constitution and broad remit, WHO can be perceived to be slow-moving, inefficient, and unresponsive to changing situations—to lack effective leadership, in other words.

In 2014–2015, the disastrous West African outbreak of Ebola brought about a watershed moment for WHO. Despite current Director-General Margaret Chan’s earlier experience in addressing the 2003 outbreak of Severe Acute Respiratory Syndrome in Hong Kong, the agency’s response to the threat of Ebola was seen to be sluggish and indecisive. Enormous disruption and a substantial toll of deaths ensued. While the crisis was exacerbated by weak country health systems, combined with a poor understanding about the pathogenesis of and necessary control measures for Ebola infection, WHO was perceived to be responsible for the erratic overall response to the outbreak. This episode has recently been discussed by Lawrence O. Gostin of the O’Neill Institute for National and Global Health Law, Georgetown University, and colleagues in *PLOS Medicine* in an overview of four Commissions, which addressed the response to the Ebola outbreak [1]. Reformation of WHO to ready it to lead responses to future health emergencies is one area of active debate.

Chan will step down from WHO on June 30, 2017 after more than a decade in the post. The process for choosing WHO’s next leader has begun, promising to be protracted and rigorous as befits the importance of the role. Factoring in the many influential stakeholders in the process of appointing Chan’s successor, however, transparency of the selection process may be one area unlikely to attract plaudits. Although too soon to speculate about the identity of WHO’s next Director-General, it is worth reflecting on what qualities an incoming leader should bring to WHO and how that person might need to conceive changes in the structure and behavior of the organization against a landscape of important and evolving threats to the health of the fast-growing global population.

Instead of electing a new Director-General, Lorenz Von Seidlein of Mahidol University, Thailand, argued that “the problems…are now so deeply ingrained that replacing the WHO with new, more appropriate organizations is the logical solution…at a fraction of current cost, free of cumbersome, archaic obligations and entitlements and [with] an ability to respond to new problems.” This viewpoint is indicative of the strength of feeling that WHO’s deficiencies have come to evoke in some of those committed to the cause of improving the health of people in low-income and middle-income countries. But this perception acknowledges that an accountable global body will always be needed to promote, set standards in, and evaluate progress toward better health for people in all countries. The next Director-General will need to heed critics of the organization and craft a process of streamlining and restructuring to produce a new WHO that is demonstrably effective in leading responses to threats to health, and efficient in doing so. As Gostin commented to *PLOS Medicine*, “WHO urgently needs a bold reform agenda to fix long-standing problems recognized by every independent group that has evaluated the Organization.” Political machinations and the enemy within, bureaucracy, are likely to impede reform. For example, WHO’s regional and country offices are seen by some as unaccountable, yet the agency of the future will need to be connected and responsive to the resources and needs of all constituent countries. As Gostin also noted, “[WHO] has failed to include civil society in its governance, unlike…newer organizations.”

WHO’s next Director-General should be a proven leader and advocate, perhaps from a low-income or middle-income country. The new recruit will be greeted by a full in-tray, and featuring prominently are likely to be the constraints imposed by WHO’s current funding mechanisms. A substantial proportion of WHO’s existing budget is earmarked for specific projects, leaving the organization with little financial flexibility to respond to unanticipated demands. However, any improved funding mechanism is likely to follow, and be dependent on, organizational reform. According to Kruk, “WHO is both essential and hamstrung…the election of the Director-General should be a moment for member countries and other funders to reflect on whether they want an implementation agency for their favored health agenda, or an independent institution with the intelligence, agility, and operational capacity to tackle the coming global health challenges.”

Above all, the incoming leader of WHO will need to be open-minded and creative. More than one of the experts we contacted emphasized the fluid nature of the threats to human health to which WHO should shape the world’s response. WHO must be able to lead responses in some areas of global health, but, in other areas, working together with more nimble and focused organizations will be pragmatic. Large-scale infectious disease outbreaks are continuing, and noncommunicable diseases, including cancer, dementia, and mental illnesses, are growing in prevalence and increasing demand for treatment and care. The resources and ingenuity of researchers and clinicians will need to be harnessed, and interventions adapted to new settings, with much greater dynamism. The secular issues of population ageing, conflict, climate change, migration, and others will produce health problems that only an organization with a global reach, responsible to all, can hope to meet. We look forward to welcoming a new leader for WHO with the energy and vision to remold the organization to meet the health needs of the world’s people and societies for the 21st century.
